# Gene expression meta-analysis in diffuse low-grade glioma and the corresponding histological subtypes

**DOI:** 10.1038/s41598-017-12087-y

**Published:** 2017-09-18

**Authors:** Siqi Wang, Feng Jin, Wenliang Fan, Fang Liu, Yan Zou, Xuehan Hu, Haibo Xu, Ping Han

**Affiliations:** 1 Department of Radiology, Union Hospital, Tongji Medical College, Huazhong University of Science and Technology, Wuhan 430022, China; 2 Department of Radiology, Zhongnan Hospital, Wuhan University, Wuhan 430071, China; 3 Department of Radiology, The First Affiliated Hospital, Inner Mongolia Medical University, Hohhot 010050, China

## Abstract

Diffuse low-grade glioma (DLGG) is a well-differentiated, slow-growing tumour with an inherent tendency to progress to high-grade glioma. The potential roles of genetic alterations in DLGG development have not yet been fully delineated. Therefore, the current study performed an integrated gene expression meta-analysis of eight independent, publicly available microarray datasets including 291 DLGGs and 83 non-glioma (NG) samples to identify gene expression signatures associated with DLGG. Using INMEX, 708 differentially expressed genes (DEGs) (385 upregulated and 323 downregulated genes) were identified in DLGG compared to NG. Furthermore, 497 DEGs (222 upregulated and 275 downregulated genes) corresponding to two histological types were identified. Of these, high expression of *HIP1R* significantly correlated with increased overall survival, whereas high expression of *TBXAS1* significantly correlated with decreased overall survival. Additionally, network-based meta-analysis identified *FN1* and *APP* as the key hub genes in DLGG compared with NG. *PTPN6* and *CUL3* were the key hub genes identified in the astrocytoma relative to the oligodendroglioma. Further immunohistochemical validation revealed that MTHFD2 and SPARC were positively expressed in DLGG, whereas RBP4 was positively expressed in NG. These findings reveal potential molecular biomarkers for diagnosis and therapy in patients with DLGG and provide a rich and novel candidate reservoir for future studies.

## Introduction

According to histological characteristics, gliomas can be classified into grades I–IV based on World Health Organization (WHO) criteria published in 2007 and 2016^1,2^. Patients with low-grade glioma (grades I and II) have a median survival time of 4.7–9.8 years, with a range of up to 13 years for certain subtypes^3,4^. Grade I gliomas are often localized and are more likely to be cured after surgical resection. Grade II gliomas, also known as diffuse low-grade glioma (DLGG), account for approximately 15% of all gliomas^1^ and have heterogeneous and complicated presentations that correspond to three histological types: astrocytoma (A), oligodendroglioma (OD), and oligoastrocytoma (OA). Okamoto *et al*.^5^ demonstrated that histological type is a significant predictor of survival of patients with DLGG. Patients with OD survive longer (median survival time: 11.6 years) than patients with A (median survival time: 5.6 years)^6^. Although DLGGs have low-level proliferative activity, their natural course is to transform or dedifferentiate into high-grade glioma (WHO grade III–IV), and they often recur after surgical resection^1,7^. The surgical and medical management of DLGG remains one of the major controversies in current neurooncology. Thus, there is an urgent need to develop novel diagnostic and therapeutic targets for DLGG.

Hartmann *et al*. investigated the prognostic relevance of four prominent molecular markers in WHO grade II gliomas, including *TP53* mutation, 1p/19q deletion, O6-methylguanylmethyltransferase (*MGMT*) promoter methylation, and isocitrate dehydrogenase 1 (IDH1) mutation. The results showed that 1p/19q codeletion and IDH1 mutation are prognostic markers following the administration of radiotherapy or chemotherapy^8^. *TP53* mutations have been most commonly identified in A, whereas 1p/19q codeletion is more common in OD. OAs appear to be heterogeneous and typically show either *TP53* mutations or 1p/19q deletion^8^. The 2016 WHO classification of CNS tumours defines tumour entities based on histology and a combination of molecular aberrations, such as IDH mutation, *ATRX* mutation, 1p/19q deletion, and *TP53* mutation^2^. As we gain further insight into molecular biomarkers of glioma, the impact of these markers on diagnosis and treatment continues to evolve.

High-throughput genomics technologies, such as microarrays that provide simultaneous measurements of the expression profiles of thousands of genes, have provided substantial insight into the processes that drive disease development. Although prior studies utilizing microarrays have identified numerous differentially expressed genes (DEGs), inconsistencies exist between studies due to variations in sample size and quality^9,10^. To address this limitation, meta-analyses have been applied to synthesize the information available in publically available gene expression datasets to identify reliable molecular biomarkers of disease^11^. Importantly, meta-analyses provide enhanced statistical power, allowing the discovery of robust and reliable gene signatures. Prior meta-analyses have been performed to investigate biomarkers in breast cancer^12^, prostate cancer^13^, liver cancer^14^, and lung cancer^15^. Integrative meta-analysis of expression data (INMEX), which allows simultaneous analysis of multiple gene expression datasets, has also been applied^16–18^.

In the present study, we used INMEX to perform meta-analyses of eight eligible microarray datasets to identify key regulators and potential diagnostic and therapy biomarkers associated with DLGG and its clinical subtypes. To the best of our knowledge, this study is the first to explore diagnostic and therapy biomarkers associated with DLGG and its histological subtypes by performing meta-analyses of gene expression datasets.

## Results

### Studies included in the meta-analysis

**Figure 1:**
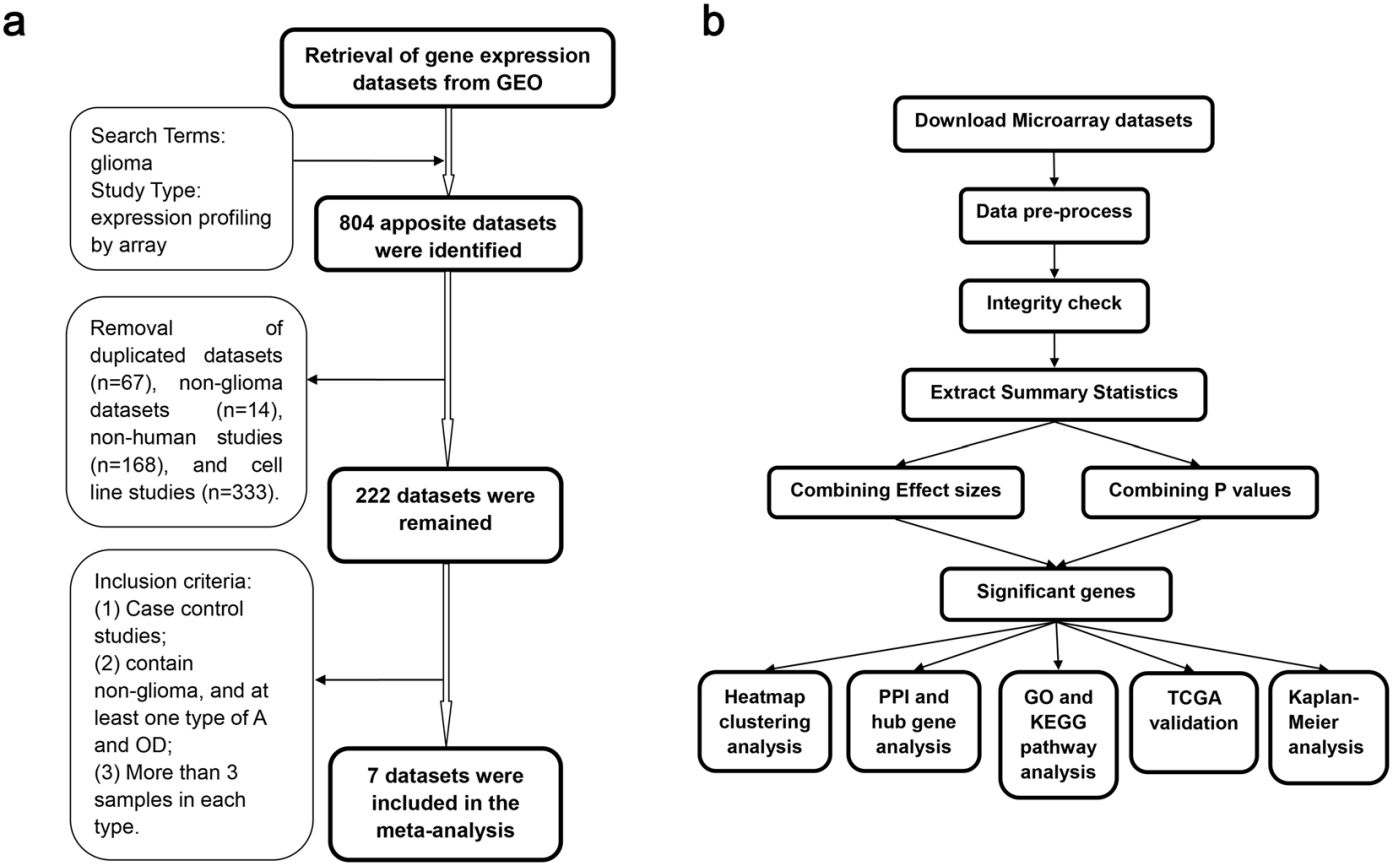
Study workflow. (**a**) Identification of eligible gene expression datasets for meta-analysis of DLGG. (**b**) The process used for meta-analysis. Abbreviations: DLGG, diffuse low-grade glioma; GEO, Gene Expression Omnibus; A, astrocytoma; OD, oligodendroglioma; PPI, protein-protein interaction; GO, Gene Ontology; KEGG, Kyoto Encyclopedia of Genes and Genomes; TCGA, the Cancer Genome Atlas.

A total of 7 studies from the Gene Expression Omnibus (GEO) dataset were included: GSE68848^19^, GSE16011^20^, GSE4290^21^, GSE12657, GSE21354^22^, GSE2223^23^, and GSE70231^24^. Additionally, mRNA expression data from 97 WHO grade II samples, including 58A, 17 OD, 22 OA, and 5 non-glioma (NG) samples, were collected from the Chinese Glioma Genome Atlas (CGGA)^25^. These eight studies were examined using meta-analysis to identify differences between DLGGs and NGs and included a total of 291 cases and 83 controls. To identify possible DEGs between histological DLGG subtypes (A and OD, but not OA, which is not recognized as a separate tumour entity in the 2016 CNS tumour classification system^2^), an additional meta-analysis was performed to examine differences between A and OD samples. For this purpose, five datasets containing information on A and OD (GSE4290, GSE16011, GSE21354, GSE68848, and CGGA) were selected to compare mRNA expression signatures among low-grade glioma subtypes; these datasets included a total of 148A and 98 OD samples. Table 1 provides detailed information on each dataset including the number of each sample type, reference, and the microarray platform used. We obtained expression data and clinical information for 254 patients from the TCGA; these data included 63A, 112 OD, 74 OA, and 5 NG samples. Figure 1 depicts our experimental workflow.

**Table 1:** Characteristics of datas.ets included in the meta-analysis of DLGG vs. NG and A vs. OG tissues. Abbreviations: DLGG, diffused low-grade glioma; NG, non-glioma; GSE, gene expression omnibus; GPL, gene platform; CGGA, the Chinese Glioma Genome Atlas; A, astrocytoma; OD, oligodendroglioma.

Source accession	Publication year	First author	Country	Platform	Numbers		Reference
Datasets included in the meta-analysis of DLGG vs. NG tissues					DLGG	NG	
GSE68848	2015	Fine H	USA	GPL570, Affymetrix U133 Plus 2.0	99	28	22
GSE16011	2010	Gravendeel LA	Netherlands	GPL8542, Affymetrix U133 Plus 2.0	21	8	23
GSE4290	2006	Fine HA	USA	GPL570, Affymetrix U133 Plus 2.0	45	23	24
GSE12657	2008	Moran LB	United Kingdom	GPL8300, Affymetrix U95 Version 2 Array	7	5	NA
GSE21354	2010	Liu Z	China	GPL570, Affymetrix U133 Plus 2.0	10	4	25
GSE2223	2006	Bredel M	USA	GPL1833, SHFK	6	4	26
GSE70231	2015	Mervi Heiskanen	USA	GPL80, Affymetrix Human Full Length HuGeneFL Array	6	6	27
CGGA	2012	Yan W	China	Agilent Whole Human Genome Array platform	97	5	28
Datasets included in the meta-analysis of A vs. OD tissues					A	OD	
GSE68848	2015	Fine H	USA	GPL570, Affymetrix U133 Plus 2.0	65	30	22
GSE16011	2010	Gravendeel LA	Netherlands	GPL8542, Affymetrix U133 Plus 2.0	13	8	23
GSE4290	2006	Fine HA	USA	GPL570, Affymetrix U133 Plus 2.0	7	38	24
GSE21354	2010	Liu Z	China	GPL570, Affymetrix U133 Plus 2.0	5	5	25
CGGA	2012	Yan W	China	Agilent Whole Human Genome Array platform	58	17	28

### Batch effect adjustment

Before performing the DLGG and subtype meta-analyses, we corrected for the batch effect using the ComBat procedure in INMEX. The principal component analysis plot showed that each dataset was clearly separated from the others before applying the batch adjustment algorithm. After the adjustment, the samples from all datasets were well intermixed as shown in Supplementary Fig. S1.

### Meta-analysis of gene expression in DLGG

**Figure 2:**
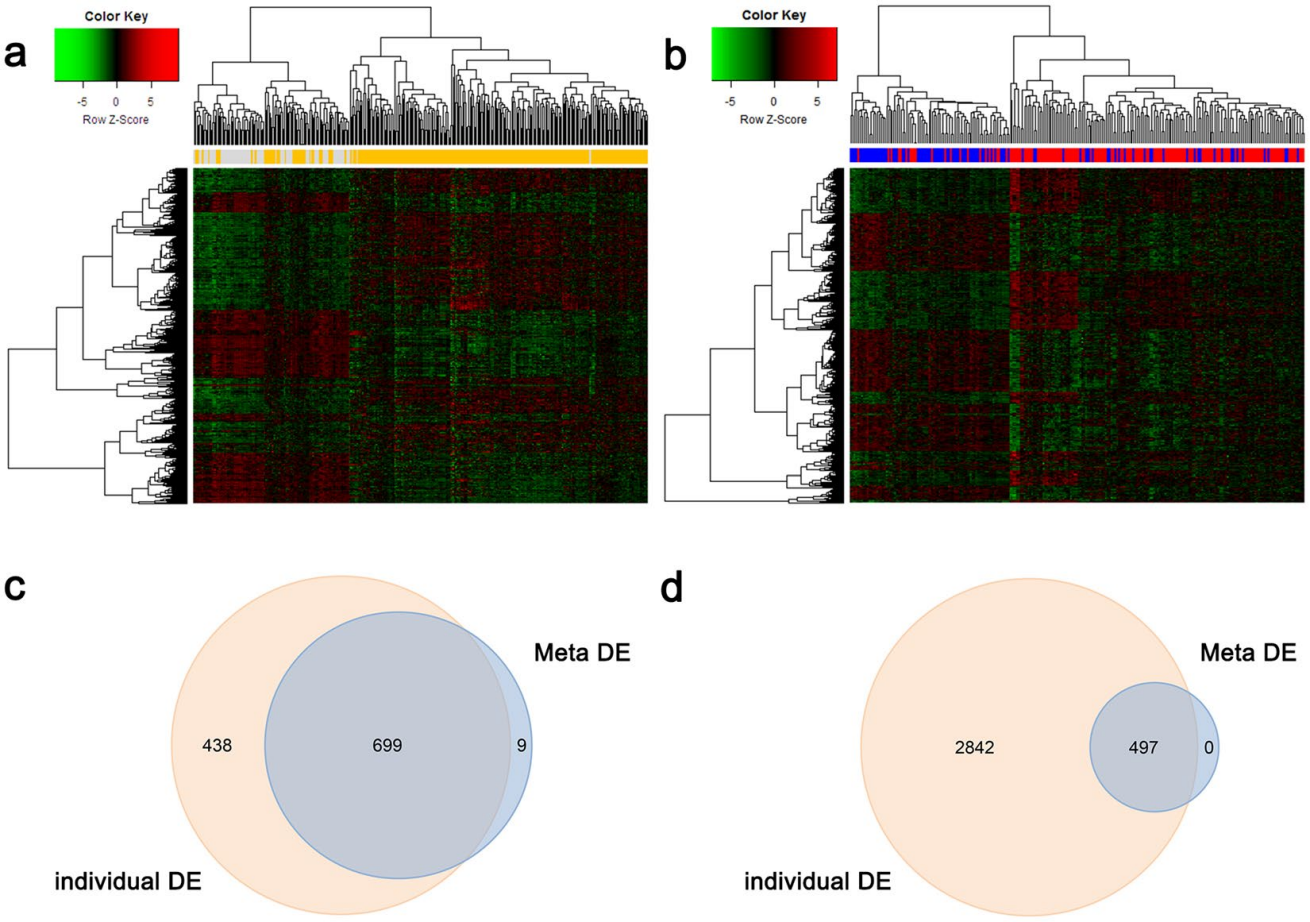
Hierarchical clustering and Venn diagram. (**a**) Two-way hierarchical clustering based on 708 DEGs in DLGG vs. NG tissues across 8 datasets. DLGG (orange label) and NG (grey label) samples fell into two major clusters. (**b**) Two-way hierarchical clustering based on 497 DEGs in A vs. OD tissues across 5 datasets. A (red label) and OD (blue label) samples fell into two major groups. (**c**) Venn diagram showing the common and unique DE genes in DLGG vs. NG tissues between the Meta-DE and Individual-DE datasets. (**d**) Venn diagram showing the common and unique DE genes in A vs. OD tissues between the Meta-DE and Individual-DE datasets. Abbreviations: DLGG, diffuse low-grade glioma; NG, non-glioma; A, astrocytoma; OD, oligodendroglioma; DE, differentially expressed.

Gained genes were defined as DEGs with weak but consistent expression profiles across all the datasets, and lost genes were identified as DEGs that appeared in individual analysis, but not in the meta-analysis, or those with large variations across different studies (due to experimental errors or different platforms). In total, there were 9 gained genes and 438 lost genes in the DLGG meta-analysis (Fig. 2c, Supplementary Table S1). From the microarray meta-analysis, we identified 708 DEGs, including 385 upregulated and 323 downregulated genes, as shown in Supplementary Table S1. We conducted two-way hierarchical clustering analysis of all DEGs. The hierarchical clustering map revealed that DLGG and NG samples were non-random partitioned into two major groups (Fig. 2a). In the DLGG datasets, secreted protein acidic and cysteine rich (*SPARC*), methylenetetrahydrofolate dehydrogenase (NADP + dependent) 2, methenyltetrahydrofolate cyclohydrolase (*MTHFD2*), and protein tyrosine phosphatase, receptor type Z1 (*PTPRZ1*) were the most significantly upregulated genes, and retinol-binding protein 4 (*RBP4*), cholecystokinin-B receptor (*CCKBR*), and syntaxin 1A (*STX1A*) were the most significantly downregulated genes. The top 10 upregulated and downregulated genes are shown in Table 2.

**Table 2:** Top 20 DEGs identified in the meta-analysis of DLGG vs. NG and A vs. OD tissues. The DEGs were ranked according to the combined effect size. Abbreviations: ES, effect size; DLGG, diffused low-grade glioma; NG, non-glioma NG; A, astrocytoma; OD, oligodendroglioma.

Entrez ID	Gene symbol	Gene name	Combined ES	Adjusted *p-value*
Top 10 upregulated genes in DLGG vs. NG				
6678	*SPARC*	Secreted protein acidic and cysteine rich	2.8727	0
10797	*MTHFD2*	Methylenetetrahydrofolate dehydrogenase (NADP+ dependent) 2, methenyltetrahydrofolate cyclohydrolase	2.8634	0
5803	*PTPRZ1*	Protein tyrosine phosphatase, receptor type Z1	2.7797	1.26E-10
6659	*SOX4*	SRY-box 4	2.6366	0
9459	*ARHGEF6*	Rac/Cdc42 guanine nucleotide exchange factor 6	2.4971	0
5375	*PMP2*	Peripheral myelin protein 2	2.4946	0
6938	*TCF12*	Transcription factor 12	2.4753	0
6175	*RPLP0*	Ribosomal protein lateral stalk subunit P0	2.4696	0
7079	*TIMP4*	TIMP metallopeptidase inhibitor 4	2.4588	0
7078	*TIMP3*	TIMP metallopeptidase inhibitor 3	2.3411	0
Top 10 downregulated genes in DLGG vs. NG				
5950	*RBP4*	Retinol-binding protein 4	−2⊡7344	6.24E-15
887	*CCKBR*	Cholecystokinin B receptor	−2⊡6242	6.24E-15
6804	*STX1A*	Syntaxin 1A	−2⊡5435	0
23531	*MMD*	Monocyte to macrophage differentiation associated	−2.5336	0
7781	*SLC30A3*	Solute carrier family 30 member 3	−2.5278	7.92E-09
1428	*CRYM*	Crystallin mu	−2.4535	0
1020	*CDK5*	Cyclin-dependent kinase 5	−2.4533	0
3761	*KCNJ4*	Potassium voltage-gated channel subfamily J member 4	−2.4439	0
2555	*GABRA2*	Gamma-aminobutyric acid type A receptor alpha2 subunit	−2.4082	0
529	*ATP6V1E1*	ATPase H+ transporting V1 subunit E1	−2.4026	0
Top 10 Upregulated Genes in A vs. OD				
51411	*BIN2*	Bridging integrator 2	1.6907	0.0000223
6916	*TBXAS1*	Thromboxane A synthase 1	1.6221	0.0000863
338773	*TMEM119*	Transmembrane protein 119	1.6106	0.000045
7462	*LAT2*	Linker for activation of T-cells family member 2	1.5738	0.0000291
81704	*DOCK8*	Dedicator of cytokinesis 8	1.5245	0.0000641
54518	*APBB1IP*	Amyloid beta precursor protein binding family B member 1 interacting protein	1.4871	0.0000329
5272	*SERPINB9*	Serpin family B member 9	1.4734	0.000000143
23533	*PIK3R5*	Phosphoinositide-3-kinase regulatory subunit 5	1.4619	0.00000757
54440	*SASH3*	SAM and SH3 domain containing 3	1.4526	0.00000121
112616	*CMTM7*	CKLF-like MARVEL transmembrane domain containing 7	1.4252	0.0000214
Top 10 downregulated genes in A vs. OD				
55140	*ELP3*	Elongator acetyltransferase complex subunit 3	−1.4435	0.0000694
3313	*HSPA9*	Heat shock protein family A (Hsp70) member 9	−1.318	8.73E-08
9026	*HIP1R*	Huntingtin interacting protein 1 related	−1.3054	2.84E-09
23219	*FBXO28*	F-box protein 28	−1.2516	0.0000275
84894	*LINGO1*	Leucine rich repeat and Ig domain containing 1	−1.2256	0.0000642
440026	*TMEM41B*	Transmembrane protein 41B	−1.2254	8.18E-11
79608	*RIC3*	RIC3 acetylcholine receptor chaperone	−1.2223	8.18E-11
51340	*CRNKL1*	Crooked neck pre-mRNA splicing factor 1	−1.2067	0.0000443
140767	*NRSN1*	Neurensin 1	−1.1524	0.0000705
8539	*API5*	Apoptosis inhibitor 5	−1.1432	1.12E-09

Meta-analysis was also used to compare the two subtypes of DLGG among five datasets. There were 0 gained genes and 2842 lost genes in the meta-analysis of the A vs. OD samples (Fig. 2d, Supplementary Table S2). In the microarray meta-analysis, a total of 497 DEGs were identified, including 222 upregulated and 275 downregulated genes, as shown in Supplementary Table S2. The two-way hierarchical clustering map in Fig. 2b shows that A and OD samples were non-random partitioned into two major groups. Bridging integrator 2 (*BIN2*), Thromboxane A synthase 1(*TBXAS1*), and Transmembrane protein 119 (*TMEM119*) were the most significantly upregulated genes, and elongator acetyltransferase complex subunit 3 (*ELP3*), and Heat shock protein family A (Hsp70) member 9 (*HSPA9*), and Huntingtin interacting protein 1 related (*HIP1R*) were the most significantly downregulated genes between the A and OD samples. The top 10 upregulated and downregulated genes are shown in Table 2.

### Identification of hub genes using network-based meta-analysis

**Figure 3:**
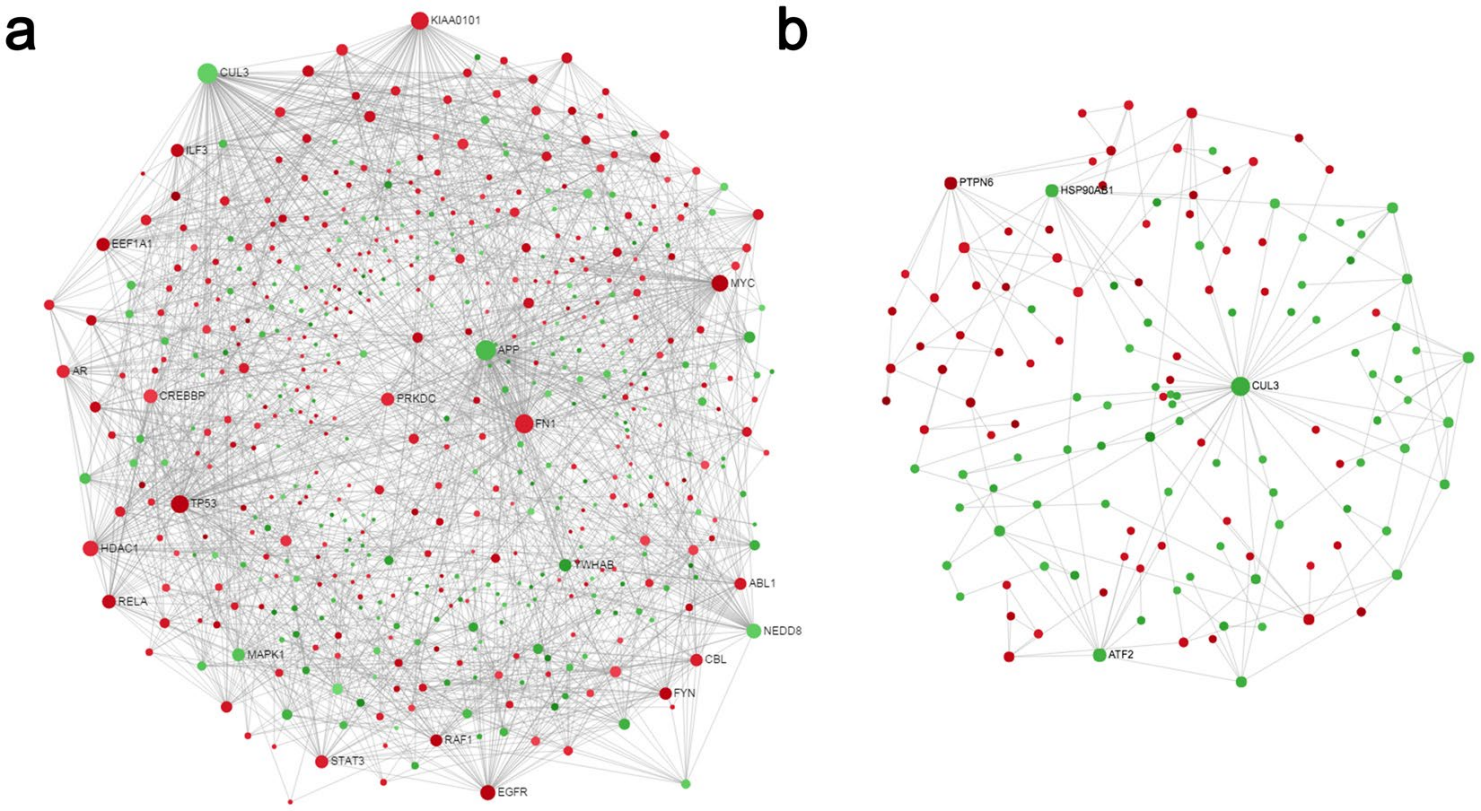
Network-based meta-analysis of hub genes. (**a**) “Zero order” interaction network of the DEGs identified in the meta-analysis of DLGG vs. NG tissues shown in a force atlas layout format. (**b**) “Zero order” interaction network of the DEGs identified in the meta-analysis of A vs. OD tissues shown in a force atlas layout format. The red and green nodes represent upregulated and downregulated DEGs, respectively. Abbreviations: DLGG, diffuse low-grade glioma; NG, non-glioma; A, astrocytoma; OD, oligodendroglioma; DEG, differentially expressed gene.

We constructed a network to identify the critical hub genes among the DEGs identified in the meta-analysis. NetworkAnalyst, available on the web, enables analysis of protein-protein interaction (PPI) networks for multiple gene lists using InnateDB. The database integrates experimental data from IntAct, MINT, BIND, BioGRID and DIP with manually curated protein interaction data from the published literature. The expanded PPI network for DLGG contained 8713 nodes and 31,253 connection edges. We conducted “Zero order” interaction network analysis in a layout format using a force atlas to better visualize the network, which included 535 nodes and 1856 connection edges (Fig. 3a). Hub genes in the network were ranked by degree. Within this network, fibronectin 1 (*FN1*), which had an adjusted *p-value* of 3.28E-15 and a combined effect size (ES) of 1.1499, was the most highly ranked hub gene (degree = 80; betweenness = 15,956.77) among the upregulated DEGs, and amyloid beta precursor protein (*APP*), which had an adjusted *p-value* of 4.55E-05 and a combined ES of −1.2673, was the most highly ranked hub gene (degree = 98; betweenness = 27455.55) among the downregulated DEGs. The top ten hub genes are detailed in Supplementary Table S1.

We also conducted network analysis to identify differences between A and OD tissues using NetworkAnalyst. The expanded PPI network for DLGG contained 4732 nodes and 9792 connection edges. The “zero order” interaction network was examined in a force atlas layout format and included 137 nodes and 187 connection edges (Fig. 3b). Within this network, protein tyrosine phosphatase, non-receptor type 6 (PTPN6), which had an adjusted *p-value* of 2.89E-05 and a combined ES of 1.3487, was the most highly ranked hub gene (degree = 11; betweenness = 2004.06) among the upregulated DEGs, and cullin 3 (*CUL3*), which had an adjusted *p-value* of 7.00E-07 and a combined ES of −0.89921, was the most highly ranked hub gene (degree = 38; betweenness = 5903.56) among the downregulated DEGs. The top ten hub genes are shown in Supplementary Table S2.

### Functional analysis

To identify the potential functions of the identified DEGs, we performed Gene Ontology (GO) and Kyoto Encyclopedia of Genes and Genomes (KEGG) pathway analyses in the Database for Annotation, Visualization, and Integrated Discovery (DAVID). The GO analysis covered the following three domains: Biological Process (BP), Cellular Component (CC) and Molecular Function (MF).

**Figure 4:**
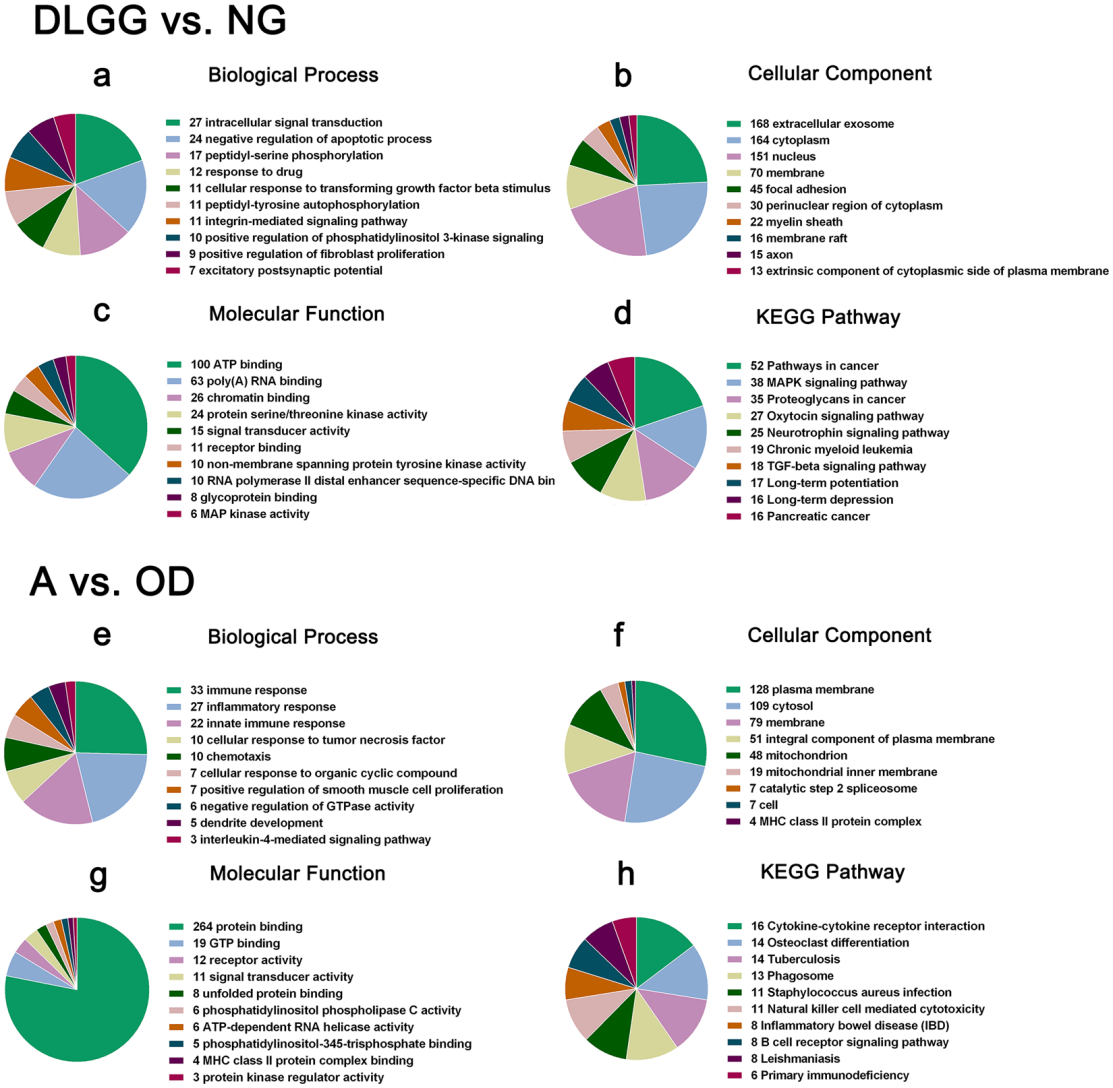
GO and KEGG pathway analysis of DEGs. The GO analysis covered the following domains: (**a**,**e**) Biological Process, (**b**,**f**) Cellular Component, and (**c**,**g**) Molecular Function. (**d**,**h**) KEGG pathway analysis. *P-value* < 0.05 was significant. Abbreviations: GO, Gene Ontology; KEGG, Kyoto Encyclopedia of Genes and Genomes; DEG, differentially expressed gene; DLGG, diffuse low-grade glioma; NG, non-glioma; A, astrocytoma; OD, oligodendroglioma.

When comparing the DLGG and NG samples, *pathways in cancer* was the top enriched KEGG pathway among DEGs. The top enriched GO terms included *intracellular signal transduction* (BP), *extracellular exosome* (CC), and *ATP binding* (MF). When comparing the A and OD samples, *cytokine-cytokine receptor interaction* was the top enriched KEGG pathway among DEGs, and *immune response* (BP), *plasma membrane* (CC), and *protein binding* (MF) were the top enriched GO terms among DEGs. The minimum ten enriched and significant GO terms and pathway terms were showed in Fig. 4 (panel 4 f showed a minimum of 9 enriched and statistically significant terms).

### The Cancer Genome Atlas (TCGA) dataset validation

**Figure 5:**
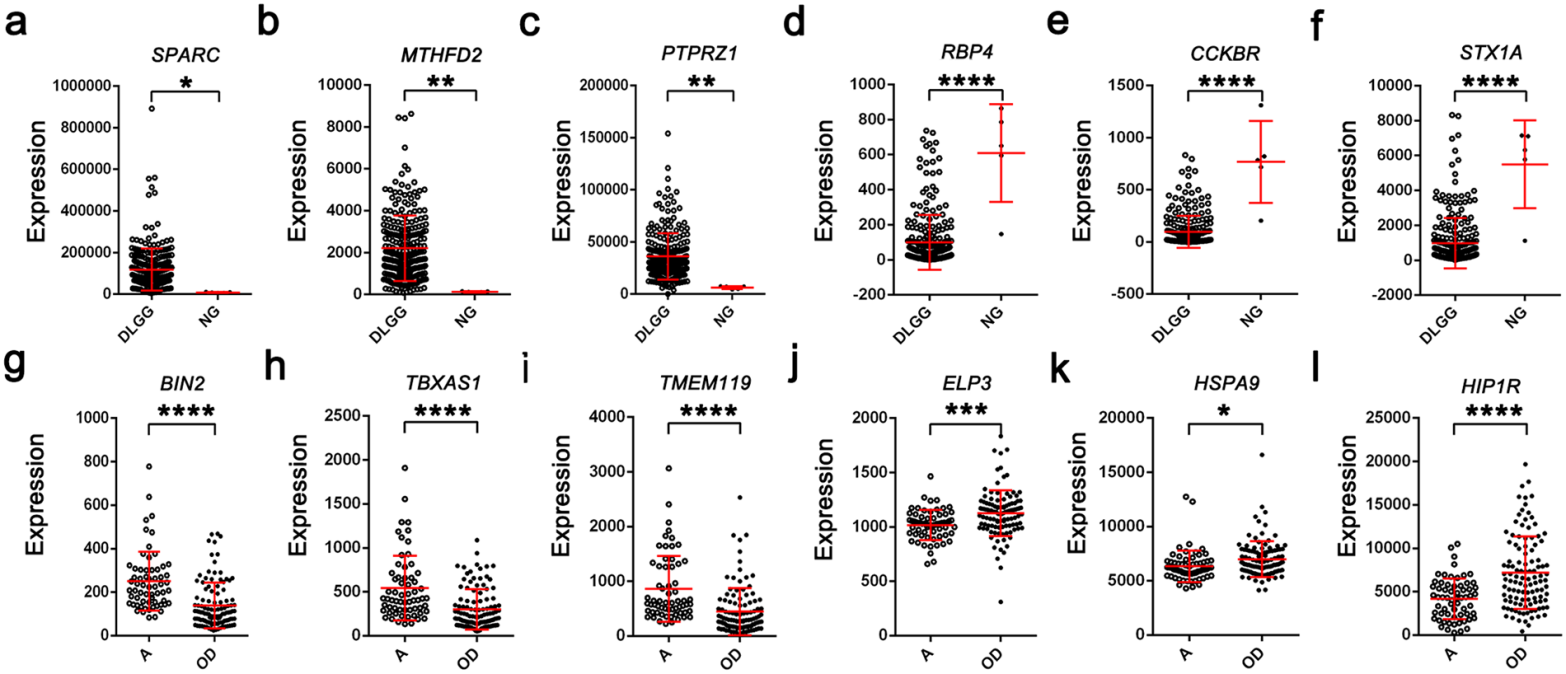
TCGA dataset validation. Expression levels of the top three upregulated (**a**–**c**) and downregulated (**d**–**f**) DEGs in the DLGG vs. NG tissues in the TCGA cohorts. Expression levels of the top three upregulated (**g**–**i**) and downregulated (**j**–**l**) DEGs in the A vs. OD tissues in the TCGA cohorts. Abbreviations: DLGG, diffuse low-grade glioma; NG, non-glioma; A, astrocytoma; OD, oligodendroglioma; DEG, differentially expressed gene. TCGA, the Cancer Genome Atlas. ****Indicates *p-value* < 0.0001; **indicates *p-value* < 0.01; *indicates *p-value* < 0.05.

The top three upregulated and downregulated mRNAs in the meta-analyses of DLGG and its subtypes were validated using the TCGA dataset. Notably, we found that *SPARC*, *MTHFD2*, and *PTPRZ1* were significantly overexpressed in the DLGG compared with the NG samples. *RBP4*, *CCKBR*, and *STX1A* were significantly underexpressed in the DLGG compared with the NG samples. *BIN2*, *TBXAS1*, and *TMEM119* were significantly overexpressed in the A samples compared with the OD samples. *ELP3*, *HSPA9*, and *HIP1R* were significantly underexpressed in the A samples compared with the OD samples (Fig. 5).

### Kaplan-Meier analysis

**Figure 6:**
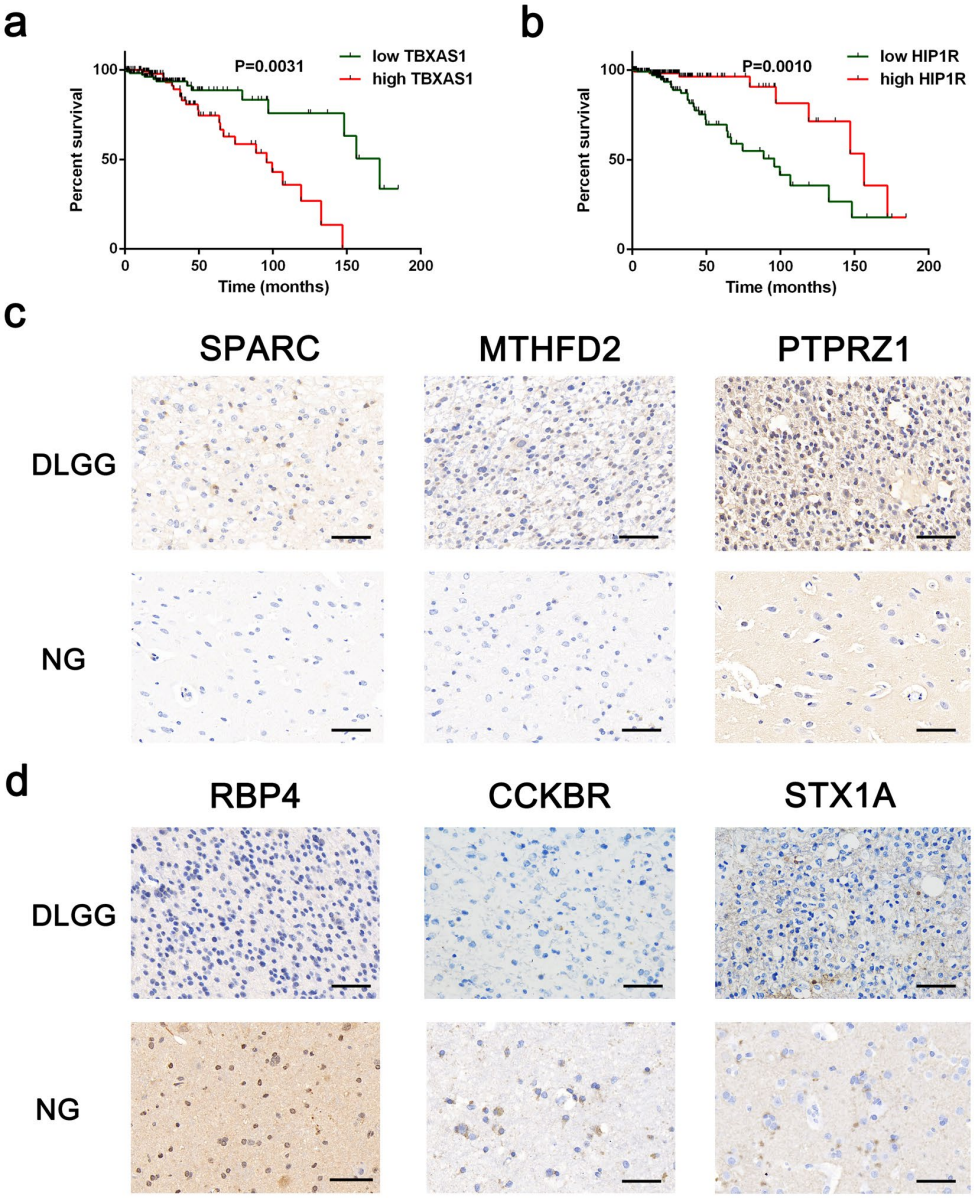
Kaplan-Meier analysis and immunohistochemistry. (**a**,**b**) Kaplan-Meier estimates of overall survival of patients with grade II gliomas. Red lines represent the high expression of DEGs and green lines represent the low expression of DEGs. The tick marks on the Kaplan-Meier survival curves represent the censored subjects. (**c**) Representative sections with immunohistochemical staining against the top 3 up-regulated DEGs in DLGG vs. NG. (**d**) Representative sections with immunohistochemical staining against the top 3 down-regulated DEGs in DLGG vs. NG. Bar = 50 μm. Abbreviations: DLGG, diffuse low-grade glioma; NG, non-glioma; DEG, differentially expressed gene.

To explore how the identified DEGs affect survival, we conducted Kaplan-Meier analysis using GraphPad Prism 6 with the DLGG cohorts in the TCGA dataset, which contained 246 patients with complete clinical traits and prognostic information. We assessed the prognostic value of the top 3 upregulated and downregulated DEGs in meta-analyses of DLGG vs. NG and A vs. OD. The results from Kaplan-Meier analysis demonstrated that patients with high *TBXAS1* expression had significantly shorter survival than those with high expression of these genes (*p* = 0.0031), and patients with high *HIP1R* expression lived significantly longer than those with low HIP1R expression (*p* = 0.0010).The Kaplan-Meier survival curves are shown in Fig. 6,a and b, detailed in Supplementary Fig. S2.

### Immunohistochemical validation

The protein expression of SPARC, MTHFD2, PTPRZ1, RBP4, CCKBR, and STX1A in DLGG and control samples were analyzed by immunohistochemistry (Fig. 6,c and d). For MTHFD2 and SPARC, weak cytoplasmic positivity were observed in DLGG samples, while control brain tissues demonstrated negative staining (*p* = 0.002 and 0.0015, respectively). High expression of RBP4 was noted in control brain tissues as against DLGG samples (*p* = 0.015). The immunohistochemical results are detailed in Supplementary Table S3.

Quantitative real-time polymerase chain reaction (qPCR) assay for the expression level of *SPARC*, *MTHFD2*, *PTPRZ1*, *RBP4*, *CCKBR*, and *STX1A* in Hs683 and HEB cells provided a further confirmation of the different expression levels of these DEGs in DLGG vs. NG (Fig. S3). Consistently with meta-analysis, the level of *SPARC* was significantly upregulated while *RBP4* and *CCKBR* were downregulated in Hs683 cells (*p* = 0.011 for SPARC, *p* < 0.0001 for RBP4, and *p* < 0.0001 for CCKBR, respectively). For *MTHFD2*, *PTPRZ1*, and *STX1A*, the qPCR results were contradicted with results in meta-analysis.

## Discussion

The 2016 version of the WHO classification criteria for glioma combined genotypic parameters with traditional histology and included IDH mutation, *ATRX* mutation, 1p/19q deletion, and *TP53* mutation as classification factors^2,26–28^. With the continued development of high-throughput genomics technology, many studies have explored genetic alterations associated with low-grade gliomas, such as telomerase reverse transcriptase (*TERT*) promoter mutation^29^, CpG island methylator phenotypes (*CIMP*)^30^, O-6-methylguanine-DNA methyltransferase (*MGMT*) promoter methylation^31^, and changes in nestin expression^32^. Although microarray studies have produced a large quantity of data, the sample sizes of most prior studies have been limited, affecting the identification of reliable DEGs. Meta-analysis of multiple microarray datasets makes it possible to increase the effective sample size and improve predictive power, making the identification of DEGs more reliable. Histological diagnosis of DLGG subtype is difficult due to inter-observer variability, particularly in cases lacking the typical symptoms associated with tumours of astrocytic or oligodendroglial lineage. In the present study, we attempted to identify DEGs associated with DLGG and its histological subtypes by performing meta-analyses of publically available microarray datasets. To the best of our knowledge, this is the first such attempt in DLGG research.

Across the eight analysed studies, we identified 708 genes that were consistently differentially expressed in DLGG vs. NG tissues (385 upregulated and 323 downregulated). Interestingly, we identified 9 gained genes with weak but consistent expression profiles across all the datasets, which were not discovered in the prior individual analyses. Among the upregulated DEGs, *SPARC* had the highest combined ES of 2.87, consistent with a report by Huang *et al*., which demonstrated that *SPARC* was upregulated by more than 2-fold in 20–60% of DLGG cases. Further immunohistochemical staining for SPARC was strongly positive in the cytoplasms of neoplastic cells and reactive astrocytes and negative in adjacent normal brain tissues^33^, which was in accordance with our immunohistochemical analysis. qPCR assay for the expression level of *SPARC* in Hs683 and HEB cells provided a further validation. *RBP4*, a recently identified adipokine, has been associated with many types of cancer. Uehara *et al*.^34^ reported that *RBP4* was over-expressed in prostate cancer cells and associated with the growth of these cells *in vitro*. In addition, higher circulating levels of *RBP4* have been associated with colon adenoma^35^, oral squamous cell cancer^36^, and breast cancer. In a study reported by Lorkova *et al*.^37^, low concentrations of *RBP4* were identified in sera collected from patients with epithelial ovarian cancer. However, no studies have investigated the relationship between *RBP4* and DLGG. *RBP4* had the highest combined ES (−2.73) among the examined downregulated DEGs, and RBP4 was negative staining in DLGG samples as against NG samples and downregulated in Hs683 cells by qPCR assay. The results implied that *RBP4* might be associated with DLGG, havig the potentail to be a potential target for diagnosis and therapy in DLGG.

*MTHFD2* is a metabolic enzyme that participates in mitochondrial folate one-carbon metabolism. Overexpression of *MTHFD2* has been associated with poor prognosis in patients with breast cancer^38^, and knockdown of *MTHFD2* in breast cancer cell lines reduced cell viability; increased apoptosis^39^; decreased migration, invasion, and metastasis^40^; and increased the expression of cancer stem cell markers^40^. Pikman *et al*. reported that knockdown of *MTHFD2* decreased cell growth, induced differentiation, and impaired colony formation in primary acute myeloid leukaemia (AML) blasts^41^. Despite the critical roles of *MTHFD2* in breast cancer and AML, the functions of this enzyme in DLGG have not been reported. The immunohistochemical results showed that MTHFD2 was positively expressed in all DLGG samples, whereas control brain tissues demonstrated negative staining. *PTPRZ1* was previously reported to be upregulated in glioblastoma compared with normal brain at both the mRNA and protein levels^42^. This finding is similar to our current results showing that *PTPRZ1* was overexpressed in DLGG vs. NG tissues. *CCKBR*, also known as *CCK2R*, is widely expressed in the human gastrointestinal tract, pancreas, and lung, as well as in some neuroendocrine tissues^43^, and may regulate cellular proliferation, differentiation, and morphology^44^. However, the role of *CCKBR* in DLGG have not been uncovered. In our study, *CCKBR* was downregulated in DLGG vs. NG and Hs683 vs. HEB cells, suggesting that *CCKBR* may be a potential supressor gene in DLGG development. *STX1A* is a member of the syntaxin superfamily, and studies have shown that the expression of this protein is correlated with Williams’s syndrome, cystic fibrosis^45^ and Alzheimer’s disease^46^. Ulloa *et al*.^47^ reported that inhibition of *STX1A* reduced glioblastoma tumour proliferation and cell invasion. In the present study, *STX1A* was downregulated in DLGG compared with control brain tissues. The association between *STX1A* and DLGG should be investigated further.

To identify possible DEGs between A and OD tissues, we performed a meta-analysis of five datasets. Across these datasets, we identified 497 genes that were consistently differentially expressed in A vs. OD tissues (222 upregulated and 275 downregulated). Among the upregulated DEGs, *BIN2*, also called *BRAP1*, had the highest combined ES of 1.69. BIN2 is a putative membrane-remodelling protein located at chromosome 4q22.1 and is primarily expressed in hematopoietic cells^48^. Some studies have reported frequent disruptions in the 4q22.1 region in breast cancer and hepatocarcinoma^49,50^. However, Kai *et al*. did not identify any important functions associated with *BIN2* in the context of hepatoma cell growth^48^. Thus, the mechanism underlying the role of *BIN2* is unclear. *ELP3* is involved in various functions, such as transcriptional elongation, tRNA modification, histone acetylation, and cell migration^51,52^. Wang *et al*.^53^ revealed that *ELP3* was significantly under-expressed in invasive ductal breast carcinoma compared to paired normal tissues. In the current study, *ELP3* had the highest combined ES (−1.44) among the downregulated DEGs in A vs. OD, suggesting that *ELP3* have the potential to function as a therapeutical biomarker of OD.

The expression levels of the top three upregulated and downregulated DEGs were also analysed using TCGA cohorts, and the results were consistent with those obtained from the meta-analysis. Moreover, Kaplan-Meier analysis revealed that high expression of *HIP1R* significantly correlated with longer overall survival, whereas high expression of *TBXAS1* significantly correlated with shorter overall survival. These findings confirm that these genes are potential prognostic biomarkers for DLGG as well as for distinguishing between astrocytic and oligodendroglial DLGG.

Network-based meta-analysis was performed on the list of DEGs to identify hub genes based on network centrality scoring. *FN1* and *APP* were the most important hub genes among the upregulated and downregulated genes, respectively, in the meta-analysis of DLGG. *FN1* encodes fibronectin, which is involved in cell adhesion and migration. Some studies have found that *FN1* is the hub gene in glioma, a result that is consistent with our findings; as such, *FN1* is a potential target for diagnosis and therapy^54,55^. *APP* mutations cause Alzheimer’s disease, and over-expression of *APP* has a link with shortened survival in patients with breast cancer^56^. Nizzari *et al*. showed that *APP* has a potential role in tumorigenesis mostly through its actions in activating extracellular signal-regulated protein kinase^57^. In the current study, *APP* was downregulated in DLGG relative to NG tissues, therefore the association between *APP* and DLGG should be explored further.

In the meta-analysis of DLGG subtypes, *PTPN6* and *CUL3* were the most important hub genes among upregulated and downregulated genes, respectively. *PTPN6* is a tyrosine phosphatase that participates in the regulation of numerous intracellular signalling cascades that modulate cell proliferation, differentiation, and apoptosis. In a report by Sooman *et al*., high *PTPN6* expression was found to contribute to worse prognosis in patients with anaplastic glioma, and there was an association between high *PTPN6* expression and worse survival in a subgroup of patients with anaplastic oligodendroglioma (*p* = 0.053)^58^. *CUL3* mediates neurofibromin destabilization, which underlies glioblastoma pathogenesis^59^. *CUL3* is also critical to the full activation of the Ras/ERK pathway, which is essential for cell proliferation, arrest, differentiation, survival, and apoptosis^60^.

According to the GO and KEGG pathway analysis, mRNAs were targeted to *pathway in cancer*, *MAPK signaling pathway*, *proteoglycans in cancer*, and *TGF-beta signaling pathway* in DLGG vs. NG tissues, which are known to contribute to tumorigenesis. Based on these findings, the GO categories and KEGG pathways identified in this study merit further study and validation.

Our study has several limitations. First, heterogeneity and confused factors may have distorted the analysis. To correct this limitation, we performed data preprocessing and normalization of each dataset as well as batch effect adjustment. Second, the samples included in the meta-analysis were relatively small, particularly for the NG tissues. The small sample size may not own enough power to detect true gene expression changes associated with DLGG. Third, we did not produce direct experimental evidence to verify the function of the identified DEGs, as the primary focus of our study was to perform meta-analyses of public datasets to discover novel or important mRNAs in DLGG and its histological subtypes. We have conducted immunohistochemical analysis and qPCR validation for six DEGs in DLGG vs. NG, however, the differences between cell lines and human tissues may lead to the contradictory results, so qPCR validation in human surgical specimens and more extensive investigations into these candidates will be performed in the future.

## Methods

### Identification of eligible gene expression datasets for low-grade glioma

We searched the National Center for Biotechnology Information (NCBI) GEO database (http://www.ncbi.nlm.nih.gov/geo/) to identify studies profiling low-grade glioma gene expression patterns. The keyword “glioma” was used for the search. Studies were included in the analysis if they met the following conditions: (1) they profiled gene expression, (2) they contained NG samples as well as at least one type of A and OD, (3) they contained more than three samples per type. We excluded studies examining non-human tissues or cell lines and studies not reporting microarray data. The following information was extracted from each identified study: GEO accession number, platform information, sample number, references, and gene expression data. We also collected DLGG samples from TCGA (https://tcga-data.nci.Nih.gov/tcga/) and CGGA (http://www.cgcg.org.cn/)^25^.

### Batch effect adjustment and individual data analysis

The batch effect correction option in INMEX^16^ (http://www.networkanalyst.ca/faces/home.xhtml) was applied to reduce potential study-specific batch effects. The ComBat procedures in INMEX were used to stabilize the expression ratios of genes with too high or too low ratios using Empirical Bayes methods and to stabilize individual gene variances by shrinking variances across all other genes^61^. Principal component analysis plots were used to visualize the sample clustering patterns before and after conducting the ComBat procedures.

### Meta-analysis of microarray datasets

The INMEX program (http://www.networkanalyst.ca/faces/home.xhtml), a web interface for integrative meta-analysis^16^, was used to conduct the microarray meta-analysis of DLGG. Every dataset was preprocessed using log2 transformation, and tables containing relative expression values were constructed showing gene information in rows and sample information in columns. Each dataset was visualized using box plots to ensure that identical distribution was present among the samples and to identify potential outliers. After uploading the datasets into the INMEX program, we annotated the data by converting different gene symbols to Entrez IDs. After all datasets were uploaded, processed, and annotated, the data integrity of each dataset was checked before proceeding to the meta-analysis stage. The meta-analysis of DLGG and NG tissues was conducted after estimating the ES to generate more biologically consistent results. We used a random effects model rather than a fixed effects model when the between-study heterogeneity based on Cochran’s Q test was significant^62^. This method is based on the moderated ES and was performed using the metaMA package^63^. The two-way hierarchical clustering analysis of DEGs were conducted by the gplots package in the R software.

### Functional analysis

To explore the functions of the identified DEGs, GO and KEGG pathway analyses were conducted using DAVID (https://david.ncifcrf.gov/). Significant GO terms and KEGG pathways were selected as the enriched terms based on values with *p* < 0.05.

### Network-Based meta-analysis

Network-based meta-analysis was carried out using NetworkAnalyst^64^. The Hub Explorer in NetworkAnalyst contains detailed information on nodes within the current network, including degree, betweenness centrality, and expression^65^. The degree was defined as the number of connections to other nodes. The betweenness centrality was the number of shortest paths going through a node. The expression was defined as the log fold change value of the corresponding node. Nodes with the highest degree or betweenness values represent the critical hubs of the network.

### Kaplan-Meier analysis

Kaplan-Meier survival curves were plotted using graphpad prism 6, which enables interactive exploration of survival correlations using the log-rank test. According to the median expression level of each DEG, we divided the patients with DLGG in the TCGA cohorts into groups with low and high DEG expression. *P* < 0.05 was considered statistically significant.

### Clinical samples and immunohistochemistry

Six DLGG samples and six NG samples were derived from patients undergoing surgical procedures at the Union Hospital of Tongji medical college, Wuhan, China. Human study protocols were approved by the ethical committee of Tongji Medical College, Huazhong University of Science and Technology in accordance with the Declaration of Helsinki. All patients in the study gave written informed consent.

Immunohistochemical analysis for SPARC, MTHFD2, PTPRZ1, RBP4, CCKBR, and STX1A on formalin-fixed, paraffin-embedded tissues were performed. Six DLGG and six NG tissues were embedded in paraffin wax and sections were deparaffinized with xylene, and rehydrated. The sections were immersed in a 0.01 mol/L citrate buffer solution at pH 6.0, and heated to repair antigen. After blocking the endogenous peroxidase activity with 3% H_2_O_2_ for 25 minutes, the sections were treated for 30 minutes with 3% bovine serum albumin. The sections were incubated with anti-SPARC antibody (1:50; Santa Cruz), anti-MTHFD2 antibody (1:50, Santa Cruz), anti-PTPRZ1 antibody (1:100; Santa Cruz), anti-RBP4 antibody (1:50; Santa Cruz), anti-CCKBR antibody (1:100; Santa Cruz), and anti-STX1A antibody (1:100; Santa Cruz), and then incubated with HRP-conjugated secondary antibodies for 50 min. The slides were counterstained with Harris’s hematoxylin, dehydrated through graded alcohols, and cleared in xylene prior to slide mounting.

### Cell culture and quantitative real-time polymerase chain reaction

The low-grade Hs683 glioma cells and normal human glial HEB cells were obtained as gifts from Prof. Yiping Li (Institute of Human Virology, Zhongshan School of Medicine, Sun Yat-sen University North Campus). These cell lines were cultured in DMEM (Hyclone) supplemented with 10% fetal bovine serum (Gibco) and 1% penicillin-streptomycin (Invitrogen) at 37 °C with 5% CO_2_.

Expression of *SPARC*, *MTHFD2*, *PTPRZ1*, *RBP4*, *CCKBR*, and *STX1A* were detected by qPCR. Total RNA was extracted from Hs683 and HEB cells by Trizol reagent (Aidlab) according to the manufacturer’ s instruction. Then 1 ug of total RNA from each sample was reverse transcribed into cDNA, which was used as the template for PCR amplification, and *GAPDH* was used as standard control. The amplification program used was performed as follows: 95 °C for 10 min, followed by 40 cycles at 95 °C for 30 sec, 60 °C for 30 sec. PCR products were visualized with ethidium bromide on 1.5% agarose gel. The primers were listed in Supplementary Table S4. The expression of each mRNA relative to *GAPDH* was calculated as 2^−[(Ct of target genes)−(Ct of GAPDH)]^.

### Statistical analysis

ES is a standardized difference that considers both the direction and the magnitude of gene expression changes. We used a random effects model of ES combination for the meta-analysis, and a stringent threshold (*p* < 0.0001) was used to identify DEGs. The Benjamini-Hochberg false discovery rate was used to correct the P values. Significantly enriched GO terms and pathways were identified using hypergeometric tests with *p* < 0.05 as the threshold value. The statistical analysis of immunohistochemical results used the fisher’s exact test (*p* < 0.05 was significant). qPCR assay for the expression levels of DEGs in cell lines were calculated using one way ANOVA analysis.

### Data availability

All data generated or analysed during this study are available from the corresponding author on reasonable request.

## Electronic supplementary material

Supplementary imformation

Supplementary Table S1

Supplementary Table S2

Supplementary Table S3

Supplementary Table S4
