# Supplementary material for: Gene expression meta-analysis in diffuse low-grade glioma and the corresponding histological subtypes
Source: Sci Rep. 2017 Sep 18;7:11741. doi: 10.1038/s41598-017-12087-y (PMC5603565; doi:10.1038/s41598-017-12087-y)
Supplement: Supplementary file 1 — Supplementary imformation [file 41598_2017_12087_MOESM1_ESM.doc]

**SUPPLEMENTARY INFORMATION**

**Gene expression meta-analysis in diffuse low-grade glioma and the corresponding histological subtypes**

Siqi Wang^1^, Feng Jin^1,3^, Wenliang Fan^1^, Fang Liu^1^, Yan Zou^1^, Xuehan Hu^1^, Haibo Xu^2^*, Ping Han^1^*

^1^ Department of Radiology, Union Hospital, Tongji Medical College, Huazhong University of Science and Technology, Wuhan 430022, China

^2^ Department of Radiology, Zhongnan Hospital, Wuhan University, Wuhan 430071, China

^3^ Department of Radiology, The First Affiliated Hospital, Inner Mongolia Medical University, Hohhot 010050, China

* Corresponding authors

E-mail: [cjr.hanping@vip.163.com](mailto:cjr.hanping@vip.163.com) (PH); [xuhaibo1120@hotmail.com](mailto:xuhaibo1120@hotmail.com) (HX)


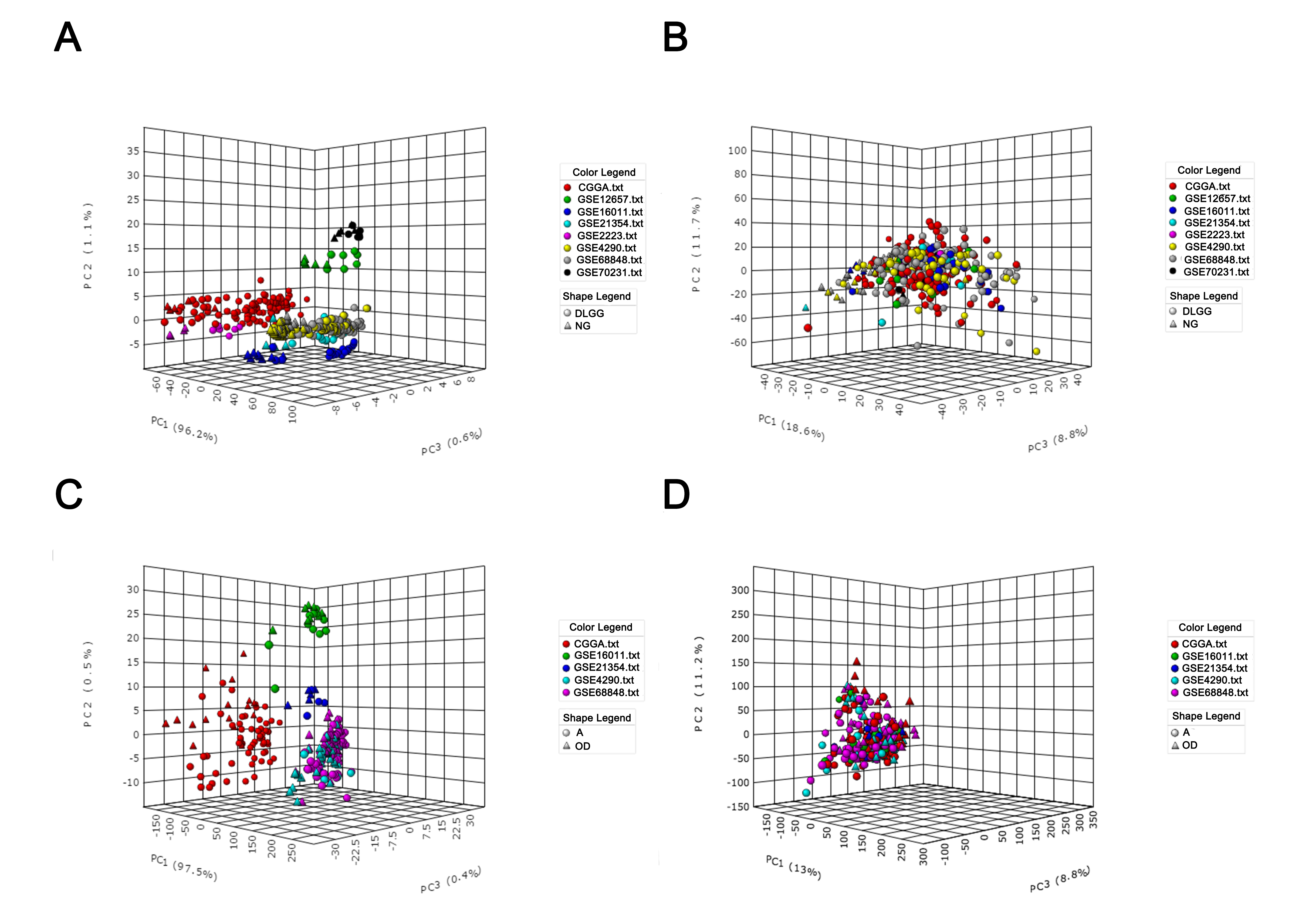


**Figure S1.** Illustration of principal component analysis plots as validation tools for batch effect removal. Plot of principal components: (**A**) before batch effect removal in DLGG vs. NG, (**B**) after batch effect removal in DLGG vs. NG (using Combat method), (**C**) before batch effect removal in A vs. OD, (**D**) after batch effect removal in A vs. OD (using Combat method). Each colour represents one dataset. Abbreviations: DLGG, diffuse low-grade glioma; NG, non-glioma; A, astrocytoma; OD, oligodendroglioma.


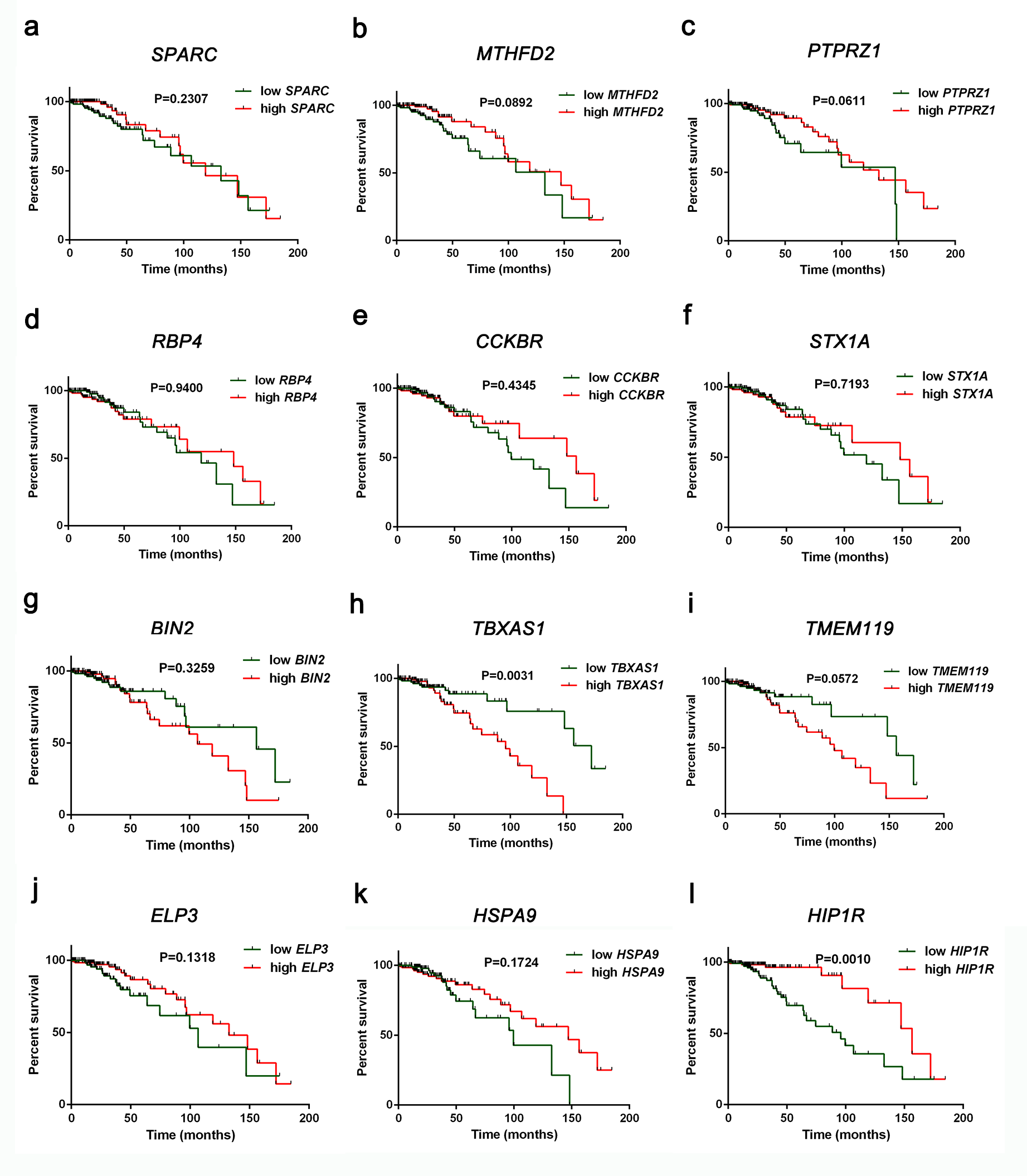


**Figure S2.** Kaplan-Meier estimates of overall survival of patients with grade II gliomas. Kaplan-Meier survival curves of : (**a**-**c**) the top 3 upregulated DEGs in DLGG vs. NG, (**d**-**f**) the top 3 downregulated DEGs in DLGG vs. NG, (**g**-**i**) the top 3 upregulated DEGs in A vs. OD, (**j**-**l**) the top 3 downregulated DEGs in A vs. OD. Red lines represent the high expression of DEGs and green lines represent the low expression of DEGs. The tick marks on the Kaplan-Meier survival curves represent the censored subjects. Abbreviations: DEG, differentially expressed gene; DLGG, diffuse low-grade glioma; NG, non-glioma; A, astrocytoma; OD, oligodendroglioma.


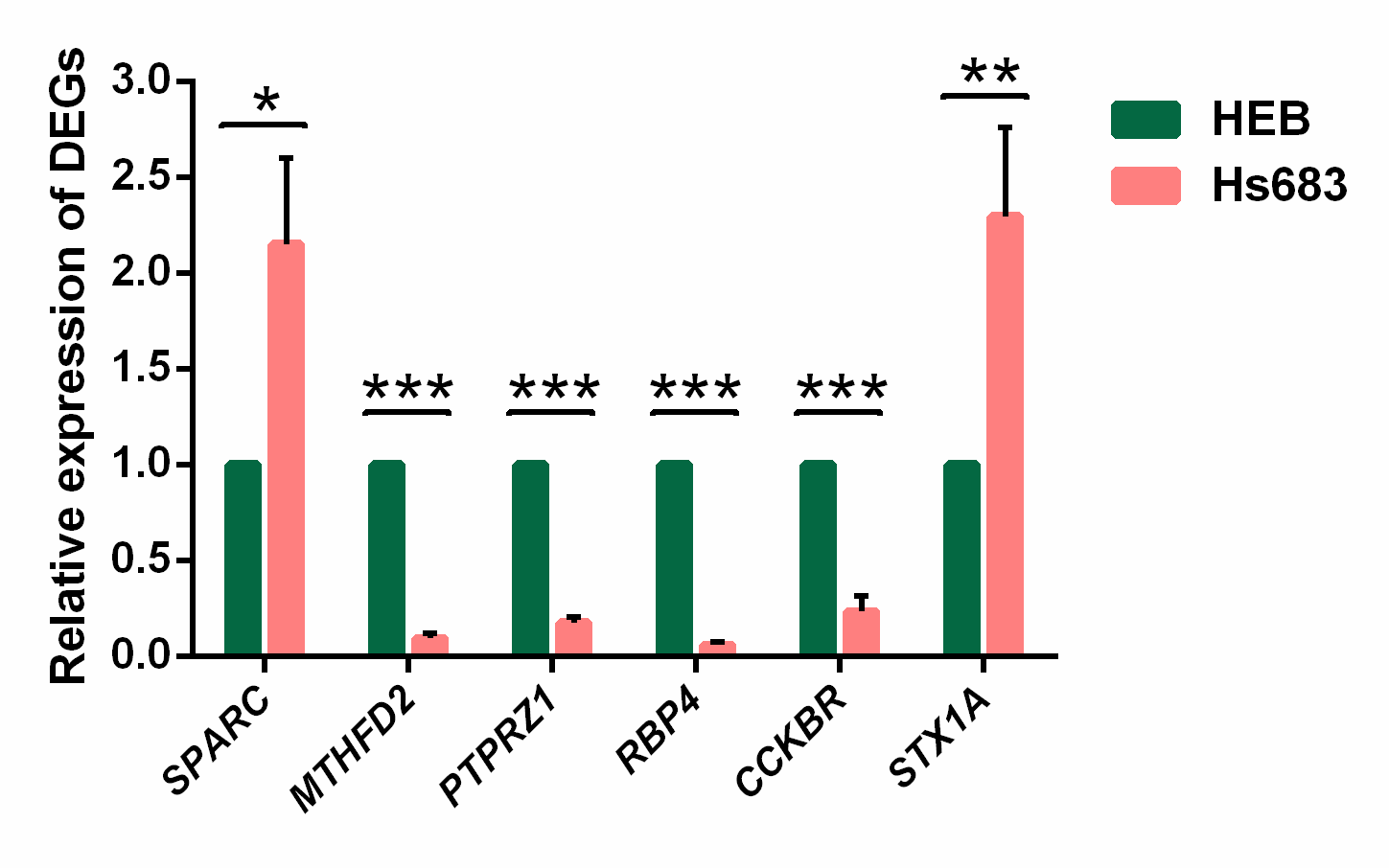


**Figure S3.** Validation of the six DEGs expression by qPCR in low-grade Hs683 glioma cells and normal human glial HEB cells. *** indicates *p-value* < 0.0001, ** indicates *p-value* < 0.01, * indicates *p-value* < 0.05, values of *p* were calculated using one-way ANOVA. Abbreviations: qPCR, quantitative real-time polymerase chain reaction; DEG, differentially expressed gene.
